# From Agro-Industrial Waste to Natural Hydrogels: A Sustainable Alternative to Reduce Water Use in Agriculture

**DOI:** 10.3390/gels11080616

**Published:** 2025-08-07

**Authors:** César F. Alonso-Cuevas, Nathiely Ramírez-Guzmán, Liliana Serna-Cock, Marcelo Guancha-Chalapud, Jorge A. Aguirre-Joya, David R. Aguillón-Gutiérrez, Alejandro Claudio-Rizo, Cristian Torres-León

**Affiliations:** 1 Center for Interdisciplinary Studies and Research (CEII), Universidad Autonoma de Coahuila, Arteaga 25280, Coahuila, Mexico; calonso@uadec.edu.mx; 2 School of Biological Sciences, Universidad Autonoma de Coahuila, Torreón 27276, Coahuila, Mexico; 3 Agri-Food and Agro-Industrial Bioeconomy Research Group, Universidad Autonoma de Coahuila, Torreón 27276, Coahuila, Mexico; 4 Faculty of Engineering and Administration, Universidad Nacional de Colombia, Palmira 763533, Colombia; lserna@unal.edu.co; 5 Centro Nacional de Asistencia Técnica a la Industria (ASTIN), Servicio Nacional de Aprendizaje (SENA), Cali 760004, Colombia; mguanchac@sena.edu.co; 6 Research Center and Ethnobiological Garden (CIJE), Autonomous University of Coahuila, Viesca 27480, Coahuila, Mexico; jorge_aguirre@uadec.edu.mx (J.A.A.-J.); david_aguillon@uadec.edu.mx (D.R.A.-G.); 7 Faculty of Chemical Sciences, Universidad Autonoma de Coahuila, Saltillo 25280, Coahuila, Mexico; jclaudio@uadec.edu.mx

**Keywords:** agro-industrial waste, agricultural hydrogels, water capability, bio compostable hydrogels, food sustainability, bioeconomy

## Abstract

The increasing demand for food necessitates that agri-food systems adopt innovative techniques to enhance food production while optimizing the use of limited resources, such as water. In agriculture, hydrogels are being increasingly used to enhance water retention and reduce irrigation requirements. However, most of these materials are based on synthetic polymers that are not biodegradable. This raises serious environmental and health concerns, highlighting the urgent need for sustainable, biodegradable alternatives. Biomass-derived from agro-industrial waste presents a substantial potential for producing hydrogels, which can effectively function as water collectors and suppliers for crops. This review article provides a comprehensive overview of recent advancements in the application of agro-industrial waste for the formulation of hydrogels. Additionally, it offers a critical analysis of the development of hydrogels utilizing natural and compostable materials. Agro-industrial and food waste, which are rich in hemicellulose and cellulose, have been utilized to enhance the mechanical properties and water absorption capacity of hydrogels. These biomaterials hold significant potential for the development of effective hydrogels in agricultural applications; they can be either hybrid or natural materials that exhibit efficacy in enhancing seed germination, improving water retention capabilities, and facilitating the controlled release of fertilizers. Natural hydrogels derived from agro-industrial waste present a sustainable technological alternative that is environmentally benign.

## 1. Introduction

The growth of the world’s population has increased the demand for food, raising concerns about the control of water use for agricultural purposes [1]. The scarcity of this resource has led to a search for new technologies that help improve agricultural performance. Hydrogels have demonstrated promising results in capturing water and enhancing water availability for plants [1]. Hydrogels are superabsorbent polymeric materials that play a vital role in addressing critical challenges within food systems, particularly in mitigating water scarcity in agricultural practices [2], nutrient management in plants [2], crop yield in the field [2], and the removal of contaminants in the soil [3].

However, commercial hydrogels are currently manufactured with materials that do not fully degrade in the environment [2]. Numerous hydrogels used in the present day exhibit prolonged degradation rates, resulting in potential accumulation in soils and aquatic environments [4]. This characteristic raises concerns regarding their long-term impact on ecosystems. The most common hydrogels are synthesized with acrylamides and acrylates that are not biodegradable [5]. Hydrogel degradation can be carried out by chemical, biological, thermal, and mechanical processes, but the degradation condition could vary by the way in which it is synthesized, the monomer used, as well as the cross-linkers and initiators [6].

In recent years, the production of hydrogels from agricultural waste has been studied [7]. The primary use of these hydrogels is to capture water from the soil [8]. Cellulose-based hydrogels can be used in various settings due to their excellent swelling capacity, water retention, and biodegradability [9].

**Figure 1:**
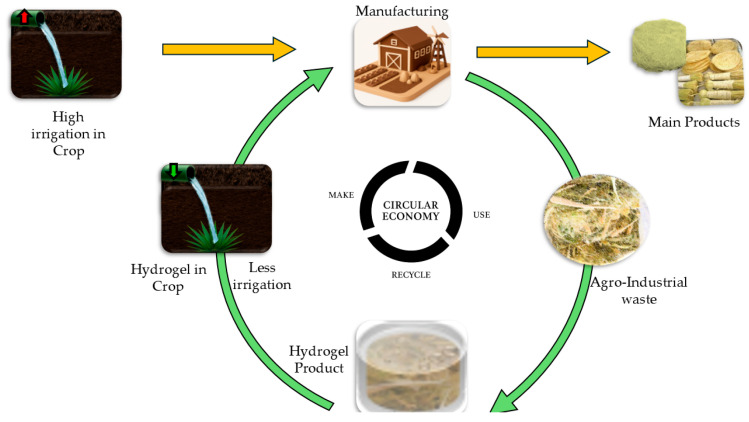
Leveraging agro-industrial waste as a model for promoting a circular bioeconomy in the processes of fiber extraction and hydrogel synthesis.

Since hydrogels for agricultural use are a technology under constant research [6,8], key aspects of their performance may vary depending on certain conditions [10], the incorporation of agro-industrial waste gives way to sustainability and the circular bioeconomy, also to the development of systems that use natural resources for subsistence, as set out in the Sustainable Development Goals (SDG) in objective number 12 [11]. Figure 1 shows the contribution to the circular bioeconomy by utilizing disposable resources and obtaining new components for incorporation into hydrogels. Selected plants are initially processed to extract primary fibers, which are used in the production of industrial goods. The residual biomass, which is traditionally discarded, is further valorized by extracting key biopolymers, including cellulose, lignin, and hemicellulose. These biopolymers, when combined with natural monomers, serve as raw materials for the synthesis of hydrogels—materials that can retain significant amounts of water. These hydrogels can then be reintegrated into agricultural systems to improve soil moisture retention, especially in arid regions. This closed-loop system exemplifies how crop by-products can be transformed into high-value functional materials, contributing to a more sustainable and circular bioeconomy.

Tariq et al. [4] previously authored an insightful and comprehensive review on the innovative development of hydrogels derived from pure biopolymers, including starch, chitosan, rubber, gelatin, lignin, and alginate. In a notable recent review, Zhu et al. [12] undertook an exhaustive examination of cutting-edge methods for producing hydrogels from natural polymers sourced from agricultural waste. However, despite the depth of these analyses, they fall short in addressing the current landscape of research focused on leveraging agroindustrial waste as a sustainable raw material for creating hydrogels for agricultural applications. Furthermore, they do not explore the remarkable potential these materials hold within a circular bioeconomy framework, a critical aspect for promoting sustainability in agriculture.

Based on the above, this review article aims to analyze the current trend in utilizing hydrogels derived from agro-industrial waste and their application as a viable alternative for water capture in agricultural areas.

## 2. Technologies and Trends in Sustainable Food Production

For decades, the way food is produced has undergone significant changes, including the improved use of fertilizers to nourish crops, the use of specialized substrates, and the conservation of water resources. The low availability of water due to droughts, resource misuse, soil erosion, and deforestation is the main problem worldwide in food cultivation [13]. Additionally, the problem is exacerbated by climate change [5], which affects security and sustainability in agri-food systems [13]. In addition, the high demand for food due to population growth has led to an increase in agricultural waste, a polluting resource [8].

Over the years, various technologies and techniques have been implemented in food production, including irrigation control, the use of fertilizers and pesticides, and soil treatment, to achieve crops with high yields [14]. The way the soil is used is also important due to its role in providing nutrients for food production. Factors such as erosion and pollution have led to the adoption of crop rotation, intercropping, and even organic farming, the latter of which is often inefficient [14].

Irrigation is a fundamental factor because water must be used efficiently. Therefore, its use varies according to soil, growing season, and crop types. Drip irrigation is a method for harnessing water, particularly in greenhouses [15], which supplies water and nutrients in a more controlled manner with minimal filtration into the soil. Food is also produced through hydroponics; this technology was developed in response to urbanization, the lack of suitable soils for cultivation, and the contamination of existing soils. Hydroponics can supply plants with essential nutrients for growth; this technique offers high yields and serves as a viable alternative to traditional agriculture [16].

Hydrogels have also been used to enhance water retention capacity and reduce irrigation frequency while also serving to aerate substrates [17]. This article focuses on an analysis of the development of hydrogel technologies that utilize agro-industrial residues in recent years.

## 3. Hydrogels in Agriculture

**Figure 2:**
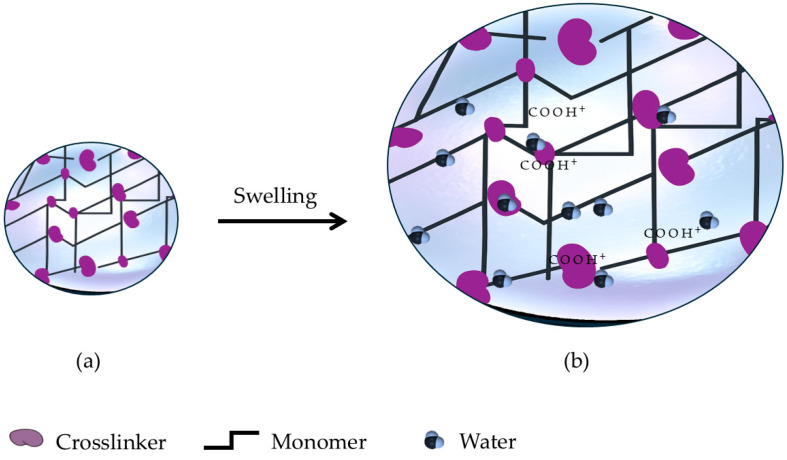
Schematic representation of a hydrogel for agricultural applications: Connections between monomers and a crosslinker (**a**) create a three-dimensional polymeric network that absorbs water molecules (**b**).

A hydrogel is a network of cross-linked polymer chains with water-retaining capacity produced by a reaction of one or more monomers. Figure 2 shows the schematic representation of a hydrogel for agricultural applications, indicating the formation of a three-dimensional polymeric network through the interaction of monomers and a crosslinking agent (a), which enables the structure to retain significant amounts of water molecules (b), a key feature for improving soil moisture retention in agricultural systems.

The hydrogels are hydrophilic and resistant to dissolution. Hydrogels can be synthesized with natural and synthetic components [18]. The use of hydrogels as substrates in horticulture has been highlighted in recent years [18]. Water retention, controlled release, and conservation are properties that are sought to be improved, in addition to the use of biopolymers that do not promote contamination [19].

The use of polymeric materials in agriculture is driven by the challenges posed by increasing food demand, but primarily by the need to utilize water more efficiently. These materials can retain and release water, promoting crop growth and soil conditioning [20]. Although hydrogels have advantages, they have also recently generated many questions: Can they biodegrade? Can they affect the soil? Can they release nano- or microplastics? Can this nano- or microplastic reach food or be deposited in the human body, causing disease? Do they have a good cost-benefit ratio? [21]. As a proposal to answer these questions, there are natural biopolymers derived from agro-industrial waste, which have a lower environmental impact. Generally, determining the environmental impact of hydrogels is based on their type, which refers to their composition, including petroleum-derived hydrogels, synthetic hydrogels, hydrogels derived from natural compounds, natural hydrogels, or hybrid hydrogels. Consequently, synthetic hydrogels generate more pollution than natural compounds.

### 3.1. Synthetic Hydrogels

Synthetic hydrogels are derived from petroleum sources and exhibit good performance in terms of mechanical and chemical properties, although they have lower biological activity [22]. Most superabsorbent hydrogels on the market are based on polyacrylamide or polyacrylate [8,10], or they are mixed with cellulose. However, concerns exist regarding their toxicity for use in agriculture or applications related to human consumption, such as their low biological activity and poor biodegradability [23].

#### Implications of Hydrogel Nanoplastics in Food Safety

In recent years, concern has grown over commercial agricultural hydrogels. Commercial agricultural hydrogels are made from synthetic polymers such as polyacrylamide and polyacrylates, which are non-biodegradable and harmful to the environment [2]. Synthetic polymers play a crucial role in enhancing soil moisture retention and minimizing irrigation requirements. However, the durability of these non-biodegradable materials poses a significant challenge, as they can break down in the environment, releasing micro- and nanoplastic particles that persist in the soil [4].

Acrylamide monomers can be absorbed through the skin and lungs, posing significant health risks, recognized neurotoxin and a potential carcinogen [24]. The absorption and accumulation of these nanoplastics in crops introduce potential contamination into the food chain, raising unforeseen risks to human health. Pinzón-Moreno et al. [25], demonstrated that synthetic hydrogels formulated from polyacrylate can generate polymeric nanoparticles, which are capable of being released into agricultural soils when exposed to water. Commercial polyacrylate-based hydrogels are not biodegradable and decompose slowly in the soil. During this degradation process, polymer fragments or nanoparticles can form, which may persist in the environment [26].

Currently, no long-term studies are proving the accumulation of commercial hydrogel nanoparticles in crops or their migration to consumers. However, existing research indicates a potential risk of soil contamination. For example, polystyrene nanoplastics have been found to accumulate in soil and be absorbed by edible crops, such as rice and peanuts, negatively impacting their nutritional quality [27]. Research has demonstrated that the small size of nanoplastics enables them to cross biological membranes and potentially cause adverse health effects [28]. Addressing the increasing concerns regarding nanoplastics in agricultural systems and their potential implications for human health presents an opportunity to advance measurement technologies and foster further research in this critical area.

### 3.2. Natural Hydrogels

Natural hydrogels are composed of natural sources, including polysaccharides and proteins. These natural resources can be derived from the agricultural sector, waste, or organisms belonging to the animal kingdom [29]. Natural biopolymers based on chitosan could be a considerable alternative to promote agricultural crop growth, as they are biodegradable and environmentally friendly [30]. One of the primary challenges for hydrogels composed of natural compounds is to identify materials with mechanical properties comparable to those of synthetic materials while maintaining biodegradability and biocompatibility [31].

### 3.3. Natural Hydrogels with Agro-Industrial Waste (Hybrids)

The global accumulation of crop residues is particularly important, as it can affect fields and contribute to pollution [32]. However, these residues contain significant amounts of cellulose and hemicellulose, which allows for compatibility with hydrogel synthesis [8]. Studies indicate that hydrogels based on various agricultural residues possess good swelling and water retention capacities, thereby improving resource availability [33].

Table 1 presents the advantages and disadvantages of various types of hydrogels based on their composition. In addition, the study objectives in hydrogels are generalized, as mentioned in the previous sections, as mechanical resistance, absorption capacity, and biocompatibility. Synthetic hydrogels are more capable of swollen water but have a negative environmental impact. Natural polymers have low mechanical properties; nevertheless, they are biodegradable and exhibit good water retention. Hybrid hydrogels share similar characteristics.

## 4. Natural Components in the Manufacture of Hydrogels

Given that natural and hybrid hydrogels exhibit superior biocompatibility, understanding the key components commonly employed in their synthesis is crucial. These components typically include polysaccharides such as starch, chitosan, alginate, and cellulose, as well as proteins like gelatin and collagen. Comprehensive information about each of these components is provided below.

### 4.1. Polysaccharides

#### 4.1.1. Starch

Starch is a natural polysaccharide composed of glucose monomers linked primarily by α-1,4-glycosidic bonds, with occasional α-1,6-glycosidic branches. Its general chemical formula is (C_6_H_10_O_5_)n, where *n* represents the number of glucose units. The molecular weight of starch varies widely depending on the source and degree of polymerization, typically ranging from 300,000 to several million Daltons. This polysaccharide possesses functional groups that enable it to form effective hydrogels, and it is both renewable and economically accessible. Starch copolymer hydrogels can be utilized in agriculture, serving as controlled-release media for pesticides and fertilizers, as well as binders for seed germination [34]. Additionally, a starch copolymer has been investigated for dye adsorption, where the adsorption capacity varies with temperature and dye concentration [35]. Starch is a useful and important component in the food industry, due to its great physicochemical properties, which allow to production of starch nanoparticles with high biocompatibility, minimal toxicity, and water dispersibility, including digestible resistant starch [36].

#### 4.1.2. Chitosan

Chitosan is a linear polysaccharide derived from the deacetylation of chitin [37]; consisting mainly of β-(1→4)-linked D-glucosamine units with varying amounts of N-acetyl-D-glucosamine. Its general chemical formula is (C_6_H_11_NO_4_)n, where *n* indicates the degree of polymerization; the molecular weight can range from 50,000 to over 1,000,000 Daltons.

Chitosan is a polysaccharide derived from chitin, one of the most abundant natural polymers after cellulose. Chitosan comes from the deacetylation of chitin through three processes: a homogeneous reaction, a heterogeneous reaction, and an enzymatic method [38]. Chitosan has a wide range of applications, including the agro-industry, where it is used in crops and post-harvest processes, as well as in the medical field, where it is utilized as a carrier for agents and dressings due to its physicochemical properties [38].

#### 4.1.3. Cellulose

Cellulose can be found in the cell walls of plants; it is considered a biopolymer of great abundance and an unlimited source of raw material. Its general chemical formula is (C_6_H_10_O_5_)n where *n* represents the number of repeating glucose units. The molecular weight of cellulose varies depending on its source and degree of polymerization, typically ranging from 100,000 to over 1,000,000 Daltons. The capacity for polymer synthesis can vary according to the source from which the cellulose is obtained and the physical and chemical treatments applied [39]. Cellulose is difficult to dissolve, and it presents complications during cross-linking. Techniques such as physical and chemical polymerization facilitate this process [40]. Hydrogels synthesized from cellulose derivatives exhibit excellent swelling, retention, and water control properties in crops [23].

### 4.2. Proteins

#### Gelatin

Gelatin, derived from collagen, is well-suited for various branches of science. This polymer is derived from the remains of mammals, including pigs and cattle [41]. Unlike polysaccharides, gelatin does not have a fixed chemical formula due to its complex protein structure. Still, it can be generally represented by the empirical formula C_102_H_151_O_39_N_31_ for an average gelatin polypeptide unit. Its molecular weight varies significantly depending on the extraction method and degree of hydrolysis, typically ranging from 20,000 to 300,000 Daltons.

In some processes, such as capsule production, there is a considerable accumulation of gelatin waste; this residue could be used for hydrogel synthesis [42]. Furthermore, it has been studied that the gelatin market is growing due to its versatility in applications, particularly in biomedical and agricultural areas [43].

Gelatin copolymer hydrogels with good mechanical properties have been synthesized, and their biodegradability has been proven [44].

### 4.3. Cross-Linkers

The use of cross-linkers is crucial in the development of hydrogels derived from natural polymers, such as gelatin, as it significantly enhances both the structural integrity and functional performance of these materials. Cross-linking agents stabilize the polymer network by forming covalent or ionic bonds between polymer chains. Among the noteworthy natural cross-linking agents are Tannic Acid, Genipin, and Citric Acid.

#### 4.3.1. Tannic Acid

Tannic acid is a natural cross-linker found in plants. Tannic acid is one of the most abundant reserve materials in plants and represents a significant source of tannins. It is available commercially, with its chemical composition primarily denoted as C_76_H_52_O_46_, corresponding to decagalloyl glucose. However, it is important to note that commercial tannic acid is generally a mixture of galloyl glucose molecules [45]. The approximate molecular weight of tannic acid is around 1701.19 g/mol. This acid is readily accessible, non-toxic, and has demonstrated its innovation in the manufacturing of biopolymers [46]. Like other natural cross-linkers, it has the advantage of being applied in different research areas. Additionally, it can enhance the physical and mechanical properties of the substances with which it is used due to the presence of hydroxyl groups in its structure [47].

#### 4.3.2. Genipin

Genipin is a naturally occurring compound extracted from the fruits *Gardenia jasminoides* and *Genipa americana*; it is used as a natural crosslinking agent in biomaterials. The chemical formula of genipin is C_11_H_14_O_5_, and its molecular weight is 226.23 g/mol. It has been used more frequently in recent studies, such as in the evaluation of the performance of genipin in the synthesis of hydrogels with chitosan, as well as evaluating its functional groups [29]. It has been reported that using genipin as a cross-linker improves the absorption capacity in media of different pH levels [48].

#### 4.3.3. Citric Acid

Citric acid is a tricarboxylic acid that occurs naturally in citrus fruits. Additionally, it can be synthesized through the fermentation of carbohydrates, including starch and glucose [49]. This compound is commonly used as a pH regulator and crosslinker in polymeric systems. Its chemical formula is C_6_H_8_O_7_, and its molecular weight is 192.12 g/mol.

In a copolymer, citric acid can increase the swelling ratio as the concentration increases [44]. Additionally, combinations of cellulose compounds have been studied and compared for their absorption behavior in the presence of citric acid, concluding that they are viable for agricultural use [50].

### 4.4. Agro-Industrial Waste as a Source of Polysaccharides

Plants are a source of polymeric compounds, and agricultural waste has been utilized to develop new technologies in recent years. These wastes are a source of cellulose and hemicellulose [9]. Once extracted, the components are used to manufacture hydrogels, which function in seed germination [51]. The following examples illustrate the utilization of agro-industrial waste as a source of natural polysaccharides to produce hydrogels.

#### 4.4.1. *Furcraea bedinghausii*

Residues from the fique plant, which belongs to the Agave family, have proven to be compatible with obtaining polymeric materials [47] and can improve the mechanical properties of hydrogels due to their cellulose content [52]. This plant is used in Colombia and is a high-production resource, producing approximately thirty thousand tons per year [52].

#### 4.4.2. *Agave tequilana* Weber

One of the plants with the highest waste production, which contains hemicellulose, cellulose, and lignin, has been utilized to develop hydrogel films. Additionally, hybrid hydrogels have been synthesized from this waste. This waste is important due to its high production and utilization [53]. *Agave tequilana* Weber, commonly known as blue agave, is primarily cultivated to produce tequila, a traditional Mexican alcoholic beverage made through fermentation and distillation of its sugars.

#### 4.4.3. *Agave lechuguilla* Torr.

This plant is a resource used to obtain the fiber called “ixtle”, which is utilized in the manufacture of brushes, construction, as well as in wickerwork and basketry [54]. Lechuguilla has been found to have potential for biotechnological applications such as the production of biofuels and chemicals with high added value, in addition to having phytochemical properties such as the content of saponins, sapogenins, phenolic compounds, and fructans, as well as lignins, cellulose, hemicellulose, and antioxidant capacity [55].

## 5. Waste-Based Hydrogels and Their Impact on Reducing Water Usage in Agriculture

The incorporation of agro-industrial residues into hydrogel formulations has demonstrated significant potential to enhance water absorption and retention.

Jong et al. [51], developed a hydrogel utilizing waste from the agro-paper industry, achieving a remarkable maximum water absorption capacity of 465.5%. This impressive result is attributed to the inclusion of 3% cellulose in the formulation. The authors also reported positive outcomes in rice germination. Hydrogels have been successfully synthesized from black liquor, a byproduct of the paper industry [56], utilizing a graft copolymerization process initiated with free radicals. Research indicates that these hydrogels exhibit a significant increase in water retention capacity, measuring at 45.25%. This enhancement is attributed to the presence of lignin and polysaccharides within the black liquor, which contributes to the formation of hydrophilic groups, such as carboxyl and hydroxyl groups, on the surface of the hydrogel. Additionally, the modification of the surface plays a critical role in enhancing the mechanical properties of hydrogels produced through bulk polymerization [18].

Guancha-Chalapud et al. [52] used nanofibers (3% *w*/*w*) from the agro-industrial waste of *F. bedinghausii* to form hydrogels using the solution polymerization method, The authors report that this hydrogel allows reducing the irrigation frequency by up to 90%. Greenhouse experiments have showcased the remarkable benefits of hydrogels crafted from agro-industrial waste abundant in polysaccharides. In a study by Madramootoo et al. [57], the use of cellulose-rich hydrogels in greenhouse tomato cultivation resulted in a 20% reduction in irrigation water—translating to a savings of 225 mm—when compared to traditional control treatments.

The agro-industrial waste of coconut fiber has also been effectively utilized in the production of hydrogels through the graft polymerization method [58]. These hydrogels possess a remarkable water absorption capacity of 342 g of water per gram of dry gel when tested in distilled water. This can potentially enhance water availability in agricultural soils by as much as 125%.

Recently, Gayen et al. [59] conducted research in which they utilized rice straw and tamarind seed residues to create hydrogels. Their findings indicated that soil incorporated with these hydrogels demonstrated a 33% enhancement in maximum water holding capacity. Furthermore, the residue from the date palm (*Phoenix dactylifera* L.) has shown significant potential in hydrogel formulation through a carboxymethylation process, followed by crosslinking with citric acid. This approach resulted in a remarkable equilibrium swelling capacity of 700% [60].

The methods of delivering water to plants vary significantly. By assessing soil moisture [51] and measuring soil electrical conductivity [56] over time, we can evaluate the availability of water for plants. A study by Sulianto et al. [61] demonstrates that a pectin-starch hydrogel can retain 62% of water in the soil after five days, whereas soil without the hydrogel shows no water availability. Furthermore, increasing the concentration of hydrogels in the soil can lead to a 125% increase in water availability [58].

These findings highlight the remarkable potential of biopolymer-rich agro-industrial waste as sustainable precursors for hydrogel synthesis. By leveraging advanced polymerization techniques, such as graft copolymerization and solution or bulk polymerization, we can unlock innovative pathways that promise both environmental sustainability and cutting-edge applications.

## 6. Trend in the Formulation of Hydrogels with Agro-Industrial Waste

**Figure 3:**
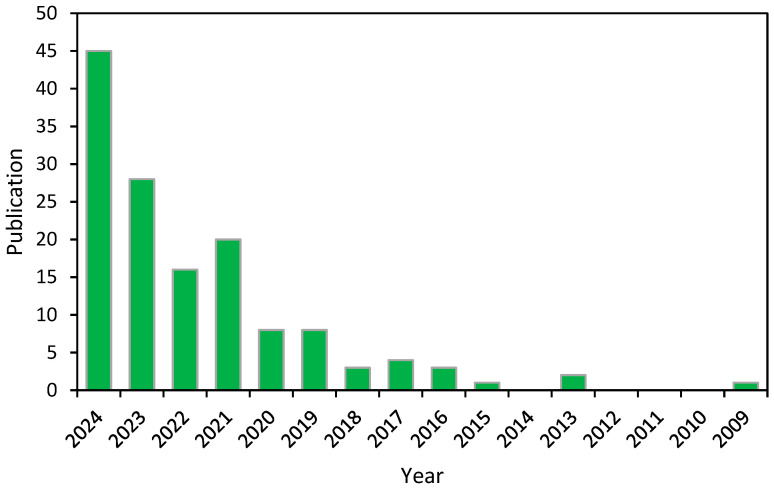
Scientific publications on hydrogels from agro-industrial and food waste sources over time: results from an advanced search in Web of Science.

As shown in Figure 3, there has been a shift in the study trend regarding the use of agro-industrial waste for hydrogel production over the past 16 years. The study of hydrogels based on agro-industrial waste for agricultural and food technology applications reveals a clear upward trend over the past decade. Between 2009 and 2015, the volume of relevant publications in the field remained low, suggesting that it was still in the early stages of development and had not yet garnered substantial academic interest during that timeframe.

Since 2016, the number of publications has steadily increased, particularly in 2019 and 2020, with eight articles published each year. From 2021 onward, this trend intensified, reaching a peak in 2024 with 45 published articles (Figure 3). This growth demonstrates that the topic has transitioned from a niche area to a recognized field of study. Continued growth is anticipated as global policies increasingly prioritize the valorization of waste materials within sustainable development initiatives.

**Figure 4:**
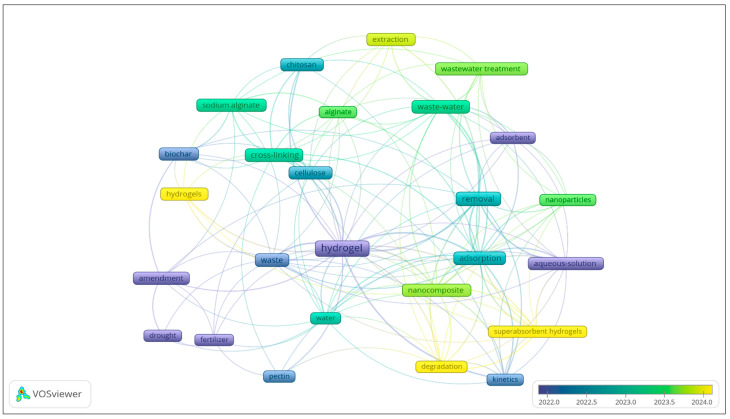
Related research trend by keywords “Based waste hydrogel” AND “Plants” from 2021 to 2024, excluding review articles, using “VOSviewer”.

Figure 4 illustrates the correlation between authorship and the number of articles on the research topic, highlighting the trend in publishing articles on hydrogels derived from natural waste. The topics of “hydrogel,” “waste,” and “water” remain relevant from 2022 to 2024. Additionally, it can be established that the keyword “hydroge*l*” predominates, with the highest number of correlation links. Additionally, it is worth noting how the words “cellulose” and “chitosan” are related to the topic “hydrogel” from 2022, which is relevant due to the use of natural components for obtaining biopolymers. The words “cross-linking” and “kinetics” reflect topics addressed in obtaining said biopolymers, using appropriate cross-linkers, carrying out extractions of chemical compounds, and testing their versatility. Also, the word “nanocomposite” and “sodium alginate,” according to the software, is the incidence of authors who worked on those topics, but the word “nanocomposite” has greater relevance according to the graph, showing a greater number of links, due to the use of nanocomposites in the synthesis of hydrogels.

**Figure 5:**
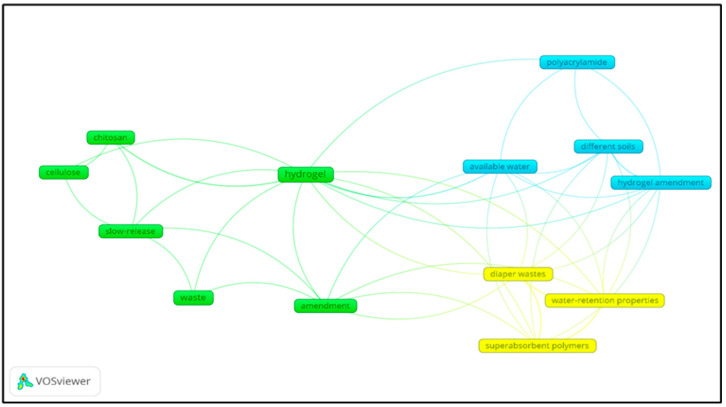
Incidence of keywords searching for “Based wasted hydrogel” AND “Plants” excluding review articles, in research areas “Agriculture and Food Science Technology”.

Additionally, Figure 5 illustrates the correlation with hydrogels, analyzing the most frequently used keywords in published articles and highlighting some possible research objectives, particularly in publications focused on Agricultural Sciences, Food Sciences, and Technology. This analysis reaffirms that the primary focus is on studying hydrogels based on natural plant residues. The word “hydrogel” is linked to three blocks denoted in different colors, representing the keywords used together for the publication of scientific articles. Figure 5 shows that the words “available water” are the second most relevant, indicating that the issue of water availability is directly related to the study of hydrogels based on agro-industrial waste.

Table 2 presents the study trend, as determined by the publication of articles, based on a systematic search using the “Web of Science” database, which defines the type of natural component used, the type of hydrogel, its application, the results found, and the authorship. Cellulose can be used as an agent in the manufacture of hydrogels. Additionally, waste from the paper agro-industry has been utilized to produce cellulose and to germinate rice seeds [51]. Guancha-Chalapud et al. report that obtaining fibers from Fique plant waste through the delignification process can be used as reinforcements of the mechanical properties of hydrogels, resulting in the improvement of hydrogels, which are capable of having greater water absorption, reducing the frequency of irrigation [52].

These residues shown in Table 2 are usable thanks to their lignin, hemicellulose and cellulose content, from which they can be extracted or not. Each residue has favored the swelling capacity of the hydrogels that have been synthesized using it. The residue of the Fique plant [52] and the residue of the coconut plant [58] have also been useful in reinforcing mechanical properties. In the case of black liquor, without performing component extraction, it helps to retain water, releasing it over a longer time. Cellulose can also be extracted from paper waste, which, when incorporated into hydrogel polymeric networks, can increase the percentage of seed germination, in addition to its performance during crop development. The importance of these residues is that they can be environmentally friendly, and this biomass can be used to generate technology for use in agriculture.

## 7. Conclusions

Various types of hydrogels have proven effective in agricultural practices, for water harvesting, fertilizer release, and as soil substrates. However, these can be improved to make them easier to manufacture. Natural hydrogels composed of components from agro-industrial waste represent a sustainable alternative with a low environmental impact. They can improve water use, reduce production costs, and are biologically friendly. The use of waste promotes a circular economy and aligns with the United Nations Sustainable Development Goals (SDGs): SDG 2, Zero Hunger, and SDG 12, Responsible Production and Consumption. Based on a review of the literature, it was found that agricultural waste has potential for the development of hydrogels. The number of publications each year demonstrates that this topic continues to gain momentum as a growing scientific research trend. Further research is needed to obtain relevant information on the use of agro-industrial waste in developing hydrogels and formulating a completely natural hydrogel with high water absorption.

## Figures and Tables

**Table 1:** Main advantages and disadvantages of hydrogel classification.

Hydrogel Classification	Advantage	Disadvantage
Synthetics	Great water retention. High wear resistance. Its manufacturing can be more controlled.	Low biodegradability. It has a negative environmental impact. Its synthesis often contaminates
Natural	Good water retention They are biodegradable Monomers are from residual natural matter	Low mechanical properties High manufacturing costs
Hybrids	Good water retention Synthetic and natural monomers are used Recyclable waste material can be used They are biocompatible	The synthesis is complex Their mechanical properties vary High manufacturing costs

**Table 2:** Trends in the Use of Agro-Industrial Waste for the Development of Polysaccharide-Based Hydrogels.

Residue	Application	Results	Polysaccharide	Swelling Capacity (%)	Authorship
Paper waste	Seed germination	Increases percentage of germination compared to soil without hydrogel	Cellulose	465	[51]
Paper waste	Tomato cultivation	They had a better response to water reabsorption	Cellulose	415	[57]
Waste from the paper industry	Water retention and controlled release of fertilizers	The values recorded for absorption capacity were better compared to the control.	Lignin	386	[56]
Fique plant residue	Reinforcing the mechanical properties of hydrogels	Increased absorption capacity	Cellulose	474	[52]
Coconut fiber	Reinforcing the mechanical properties of hydrogels	Good reswelling capacity	Cellulose and lignin	342	[58]
Rice straw and Tamarind seeds	Reinforcing mechanical properties and nutrient releaser	Great swelling capacity, long release nutrient	Cellulose	7722	[59]
Date Palm rachis	Seed germination, polymer component	Increases swelling capacity as germination seed	Lignin and Cellulose	777.8	[60]
Orange, apple, and banana peels	water content in sandy soils	High swelling capacity and increase in water content in sandy soils up to 12 days	Pectin and Starch	400	[61]

## Data Availability

Data are contained within the article.
